# PTRH2: an adhesion regulated molecular switch at the nexus of life, death, and differentiation

**DOI:** 10.1038/s41420-020-00357-0

**Published:** 2020-11-12

**Authors:** Austin D. Corpuz, Joe W. Ramos, Michelle L. Matter

**Affiliations:** 1 Cancer Biology Program, University of Hawaii Cancer Center, Honolulu, 96813 HI USA; 2 Cell and Molecular Biology Graduate Program, John A. Burns School of Medicine University of Hawaii at Mānoa, Honolulu, HI 96813 USA

**Keywords:** Apoptosis, Tumour-suppressor proteins, Growth disorders

## Abstract

Peptidyl-tRNA hydrolase 2 (PTRH2; Bit-1; Bit1) is an underappreciated regulator of adhesion signals and Bcl2 expression. Its key roles in muscle differentiation and integrin-mediated signaling are central to the pathology of a recently identified patient syndrome caused by a cluster of *Ptrh2* gene mutations. These loss-of-function mutations were identified in patients presenting with severe deleterious phenotypes of the skeletal muscle, endocrine, and nervous systems resulting in a syndrome called Infantile-onset Multisystem Nervous, Endocrine, and Pancreatic Disease (IMNEPD). In contrast, in cancer PTRH2 is a potential oncogene that promotes malignancy and metastasis. PTRH2 modulates PI3K/AKT and ERK signaling in addition to Bcl2 expression and thereby regulates key cellular processes in response to adhesion including cell survival, growth, and differentiation. In this Review, we discuss the state of the science on this important cell survival, anoikis and differentiation regulator, and opportunities for further investigation and translation. We begin with a brief overview of the structure, regulation, and subcellular localization of PTRH2. We discuss the cluster of gene mutations thus far identified which cause developmental delays and multisystem disease. We then discuss the role of PTRH2 and adhesion in breast, lung, and esophageal cancers focusing on signaling pathways involved in cell survival, cell growth, and cell differentiation.

## Facts

- PTRH2 is a bi-functional protein with pro-survival and anoikis functions.
- PTRH2 regulates adhesion mediated pro-survival signaling by upregulation of Bcl2 transcription.
- Loss of adhesion promotes PTRH2-mediated anoikis by promoting PTRH2 translocation to the cytoplasm and interaction with the Gro/Tle transcriptional co-repressor.
- Loss of PTRH2 induces premature skeletal muscle differentiation through increased caspase-3 activation.
- Congenital *Ptrh2* gene mutations cause multisystem disease including a congenital myopathy.

## Open questions

- Is PTRH2 the executioner for adhesion-dependent regulation of anoikis?
- How does cellular localization impact PTRH2 bi-functional roles?
- Is the enzymatic activity of PTRH2 required for any of these functions?
- In cancer, what is the context that determines whether PTRH2 is pro-survival or anti-metastatic?

## Introduction

**Fig. 1:**
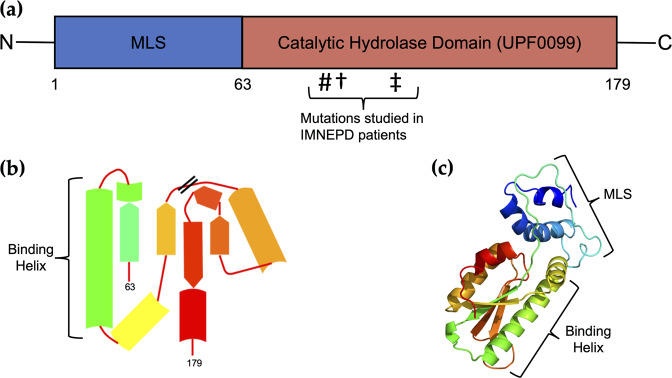
PTRH2 structure. **a** PTRH2 is 179 residues in length and contains a mitochondrial localization sequence (MLS) and a hydrolase domain (UPF0099). ^#^Missense glutamine to proline mutation of the 85th residue (p.Q85P). ^†^Frameshift mutation at the 90th residue (p.Ala90fs). ^‡^Nonsense mutation at the 108th residue (p.W108*). **b** PTRH2 catalytic domain folds into an α/β structure with a binding helix. **c** 3D structure of PTRH2 generated by X-ray crystal diffraction and visualized in Phyre2^86^.

Peptidyl-tRNA Hydrolase (PTRH2; Bit1) is a nuclear-encoded, 19 kDa protein of 179 residues located at the plasma membrane, mitochondria, endoplasmic reticulum, and Golgi apparatus in eukaryotic cells^1–4^. The relevance of PTRH2 in human disease was identified after its homologs were characterized in microorganisms. Bacterial and archaeal peptidyl-tRNA hydrolases are biologically essential as they maintain the efficiency of translation turnover by hydrolyzing aberrant peptidyl-tRNA. This catalytic activity is conserved in yeast, bacteria, and eukaryotes, although it is nonessential in yeast^5^. In humans, the *PTRH2* gene is on chromosome 17 and is expressed in all tissue types^6,7^. The 3D structure of PTRH2, as determined by 2 Å resolution crystallography, is an α/β domain consisting of a five-strand beta-sheet flanked by two alpha-helices on either side (Fig. 1)^1^. PTRH2’s crystallographic asymmetrical structure suggests it may bind to proteins via the largest alpha-helix located within its catalytic domain^1^. Indeed, PTRH2 mutations have been identified in patients suffering from Infantile-onset Multisystem Nervous, Endocrine, and Pancreatic Disease (IMNPED) that are clustered within this catalytic domain^8–12^. PTRH2 regulates cell functions including cell survival and death, muscle differentiation, and cancer cell metastasis.

The study of *PTRH2* loss of function mutations in IMNEPD patients and in a global knockout mouse has revealed its essential roles in the regulation of pro-survival and anoikis signaling in addition to cellular stress responses, cell size, and myoblast differentiation. PTRH2’s functions are determined by its cellular localization, binding partners, phosphorylation state, and integrin-mediated attachment. In cells attached to the extracellular matrix (ECM), PTRH2 mediates stress resistance through an integrin-Bcl2 pro-survival signaling pathway^3,13,14^. Conversely, in detached cells, PTRH2 is localized to the cytoplasm where it binds to the Amino-terminal Enhancer of Split (AES; also known as Transducin-Like Enhancer family member 5; TLE5) to initiate anoikis^2^. Anoikis is programmed cell death (apoptosis) that occurs when cells lose integrin-mediated attachment to the ECM^15^. Anoikis is important in normal embryonic development and is suppressed in detached cancer cells^16^. It occurs in cells and tissues where specific ECM adhesion is required for cell survival and where the loss of this adhesion inactivates cell survival pathways and instead activates a signal transduction cascade leading to activation of caspases and cell death. How cell attachment suppresses anoikis is not completely understood; however, anti-apoptotic Bcl2 family members block anoikis^17,18^. In vitro, functional analysis of PTRH2 found the catalytic domain region increases cell survival whereas a peptide fragment within the first 62 amino acids of the mitochondrial localization sequence (MLS) promotes cell death due to anoikis^19^. Because of its ability to initiate anoikis in detached cells, in part by inhibiting transcription via an unknown mechanism, PTRH2 is also referred to as Bcl2 inhibitor of transcription (Bit-1; Bit1)^2^.

Thus, the function of PTRH2/Bit1 in regulating cell survival or anoikis is context-dependent and related to the adhesion state of the cell and may further relate to the specific cell type. These roles are central to PTRH2 function in IMNEPD and cancer. Due to its context-dependent effects on cell survival and cell death, the ablation of PTRH2 in different cancers may either enhance or suppress malignancy. By summarizing current knowledge of PTRH2 and its essential roles in development, homeostasis, and metastasis, we hope to inspire further research of its potential as a therapeutic target in the treatment of congenital diseases and cancer.

## IMNEPD syndrome reveals the significance of PTRH2 in development and homeostasis

Analysis of exome and genome data from the Genome Aggregation Database version 2 (gnomAD v.2.1.1) indicates deleterious mutations of PTRH2 are extremely rare and selected against with allele frequencies on the order of 0.0004%^20^. Due to PTRH2’s recessive inheritance pattern, it is no surprise that IMNEPD is extremely rare in normal populations and has only been documented in children born to consanguineous mutant allele carriers^8–12^. Despite its rarity, studies of this disease and the individuals affected by it are important because they identify multisystemic manifestations caused by PTRH2 gene mutations indicating important roles for the protein and potential therapeutic applications.

### Three PTRH2 loss of function gene mutations manifest as disease phenotypes affecting multiple tissues

Whole-exome sequencing of consanguineous families and screening for deleterious genes was instrumental in the discovery of recessive PTRH2-null mutations that cause IMNEPD^8,9,11^. This method is highly effective for the identification of mutations that are rare in human populations due to significant negative selection. Hu et al. utilized whole-exome sequencing to identify a mutant PTRH2 allele in two children of consanguineous heterozygous carriers of Yazidian–Turkish descent^8^. This mutation (p.Ala90fs; Tables 1 and 2) consists of a CT deletion at positions 269 and 270, respectively. The resultant frameshift mutation yields a truncated 102-residue PTRH2 variant that lacks a catalytic domain, resulting in a non-functional protein. While this mutation does not alter PTRH2 mRNA transcript levels, there is decreased total PTRH2 protein in fibroblasts isolated from affected patients compared to age-matched controls^8^. Le et al.^12^ identified three patients born to first cousins of Syrian descent. These patients harbored another truncated PTRH2 variant due to a point-nonsense mutation (c.324G>A) that creates a stop codon at residue 108 (p.W108*; Tables 1, 2).

**Table 1:**
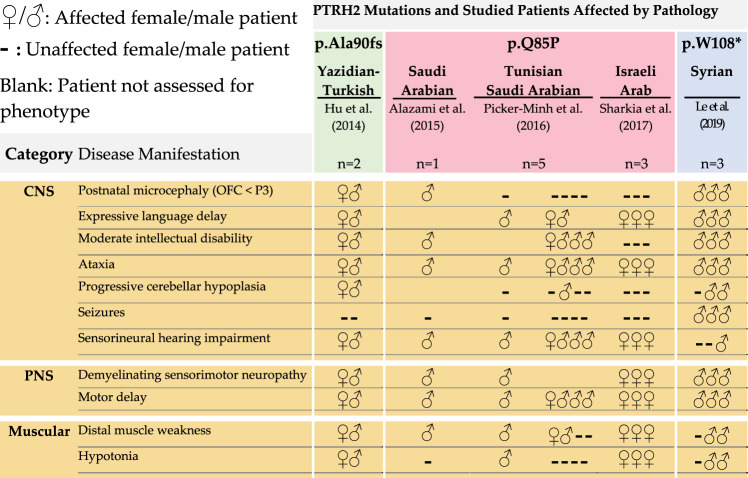
IMNEPD: neuromuscular pathology.

**Table 2:**
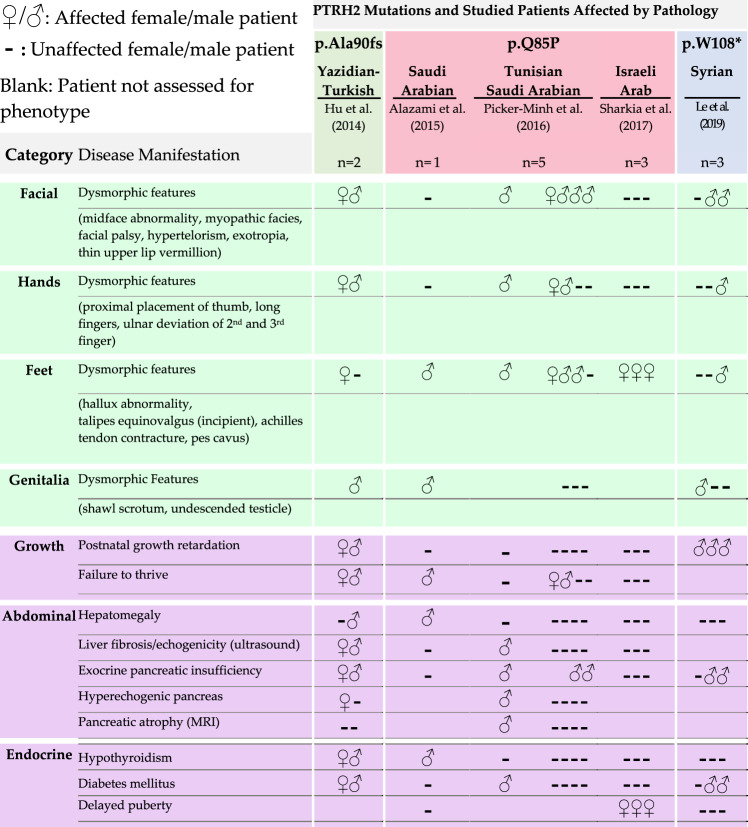
IMNEPD: physiological and developmental pathology.

Whole-exome sequencing was later used to screen the autozygome of 143 consanguineous families in which multiple members presented with similar neurogenetic phenotypes. Relevant to IMNEPD, intellectual disability was the most prevalent phenotype; however, other relevant phenotypes including ataxia and motor delay were also documented. A third mutant variant of PTRH2 was identified among a list of 68 other recessive genes associated with the neurogenetic disease. This PTRH2 variant results from a missense, glutamine to proline substitution that eliminates a hydrogen bond and introduces a kink within an alpha-helix (p.Q85P; Tables 1 and2). This mutation is proposed to destabilize the structure and function of the catalytic domain^9^. Later studies have reported this mutation and its phenotypes in eight other children from three different consanguineous families. Similar to the truncated variant, isolated patient fibroblasts with this missense mutation displayed significantly decreased PTRH2 protein levels compared to controls; yet no differences in mRNA levels^10,11^. These findings reinforce the loss of function nature of PTRH2 in the etiology of IMNEPD.

All three PTRH2 mutations manifest postnatally with similar disease phenotypes despite variations in expression and severity (Table 2). Patients harboring these PTRH2 gene mutations experienced no complications at birth and were born within the normal height and weight ranges. However, the 102-residue p.Ala90fs mutant yielded the most severe phenotypes, manifesting congenitally as distinctive dysmorphisms of the face and limbs, EEG abnormalities, microcephaly, neonatal hypotonia, stunted postnatal growth, and failure to thrive^8^. The patients harboring the nonsense mutation p.W108* shared some of these phenotypes with the addition of seizures^12^. Ultrasound revealed increased echogenicity of the liver and fibrosis of skeletal muscle while Magnetic Resonance Imaging showed cerebellar atrophy and microencephaly. Furthermore, analysis of patient blood samples indicated metabolic disorders in the form of hypothyroidism, mild diabetes mellitus, and exocrine pancreas insufficiency^8,10^.

While these phenotypes were absent or scarce in the patients with the missense mutation p.Q85P, progressive phenotypes common to PTRH2 mutations include developmental delays of speech and motor ability, demyelinating sensorimotor neuropathies, ataxia, distal muscle weakness, sensorineural hearing loss, and intellectual disability (Table 1). Importantly, motor and speech delays and signs of demyelinating sensorimotor neuropathy were reported in all patients presenting with a PTRH2 gene mutation^8–12^.

### Ptrh2-null mouse model recapitulates IMNEPD and provides new functional insight

The study of IMNEPD cases is thus far limited to a small patient cohort of 13 patients from five families. In studying children of consanguineous families, it is possible that some of the reported phenotypes may have been caused by the homozygous inheritance of other recessive disease-associated genes. This is a potential explanation of the variation of phenotypic appearance and severity reported among patients. Additionally, the young age range of the patients (3–27 years of age) at the time of study prevents investigators from making conclusions about phenotypes that may manifest later in life.

Similar to human patients, *Ptrh2*-null mice are born equal in size to age-matched wild type littermates but develop disease phenotypes postnatally. At seven days post-birth, *Ptrh2*-null mice are significantly smaller than age-matched littermate controls and display ataxia and muscle weakness and die within 2 weeks after birth. Similar to patients, *Ptrh2* knockout mice present with atrophy and hypoplasia of the cerebellum along with underdeveloped pancreas and liver parenchyma^8^. They have smaller glomeruli and muscle fibers compared to age-matched littermate controls and increased ERK activity^21^. Further analysis of these mice will enhance our understanding of the mechanisms by which PTRH2 causes IMNEPD.

## PTRH2 functions at the axis of cell survival and cell death

### PTRH2 confers stress resistance protection through pro-survival signaling in ECM-attached cells

**Fig. 2:**
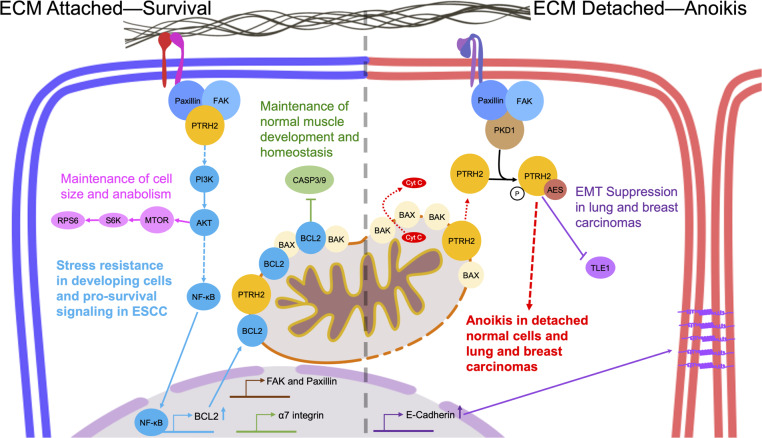
PTRH2 is involved in diverse signaling pathways depending on its cellular localization. In cells attached to the ECM by integrins, PTRH2 complexes with FAK at the cell membrane to initiate a PI3K signaling pathway to regulate homeostasis, development, stress resistance, and promote cell survival. In cells not bound to the ECM by integrins, PTRH2 is released from mitochondria into the cytosol where it complexes with AES/TLE to induce anoikis and suppress EMT.

PTRH2 was identified in an expression cloning screen to alter the transcription and protein levels of the apoptosis regulator B-cell lymphoma-2 (Bcl2) in response to integrin-mediated adhesion^2,3,22^. Griffiths et al. demonstrated PTRH2 regulates pro-survival signals in cells bound to the ECM by integrins. They reported knockdown of endogenous PTRH2 increased staurosporine-induced mitochondrial apoptosis in a variety of cell types and PTRH2 re-expression rescued these cells^3^. Upon integrin ligation, FAK is recruited to focal adhesions to induce signaling pathways that include activation of PI3K and ERK Kinase. Integrin recruitment and activation of FAK blocks apoptosis in a number of cell types^23^. FAK mediates integrin-activated bcl2 transcription^13^. Griffiths and colleagues mapped the signal transduction pathway activated by PTRH2. They found that expression of FRNK (a dominant-negative form of FAK that blocks integrin-mediated activation of FAK) abrogated the activation of *Bcl2* by PTRH2. Moreover, *Bcl2* upregulation by PTRH2 requires PI3K as cells expressing activated PI3K (p110-CAAX) demonstrated significantly increased *Bcl2* transcription and protein expression whereas expression of dominant-negative PI3K (p85D) blocks PTRH2-induced *Bcl2* transcription. Furthermore, knockdown of PTRH2 prevents activation of AKT and Bcl2 expression in adherent cells by inhibiting. NFkB translocation to the nucleus where it activates NFkB-dependent transcription of genes including *Bcl2*^24^. Indeed, in cells attached to the ECM, PTRH2 expression increased PI3K-AKT and subsequent *Bcl2* transcription. Taken together, PTRH2 promotes integrin-mediated cell survival through a PTRH2-FAK-PI3K-AKT-NFkB pathway that upregulates *Bcl2* transcription and protein expression (Fig. 2)^3,13,14^.

The loss of this PTRH2 pro-survival pathway is a key characteristic in cells isolated from IMNEPD patients. Indeed, patient fibroblasts are more susceptible to staurosporine-induced mitochondrial apoptosis compared to age-matched healthy controls. In these patient fibroblasts re-expression of wild type PTRH2 restores activation of a FAK-PI3K-AKT-NFκB-Bcl2 pro-survival pathway^8^.

### PTRH2 initiates cell death by interacting with cytoplasmic GRO/TLE

Under detached conditions, PTRH2 is released from mitochondria and presumably focal adhesions into the cytosol where it forms a complex with AES/TLE5, a dominant-negative member of the Groucho/TLE family of transcriptional corepressors, to induce cell death (Fig. 2). In adherent cells when PTRH2 is artificially targeted to the cytoplasm it binds to AES to induce cell death^2,25^. As a transcriptional co-repressor, AES binds Nuclear Factor Kappa B (NF-κB) in the nucleus to turn off transcription of NF-κB-regulated genes^26^. Although the molecular mechanism of PTRH2-mediated cell death has not been elucidated, Jenning and colleagues hypothesize that PTRH2 retains AES/TLE1 in the cytoplasm keeping TLE1 out of the nucleus and turning off AES and TLE1’s pro-survival activity^27^. Indeed, artificially overexpressing cytoplasmic PTRH2 retains TLE1 in the cytoplasm in an AES-dependent manner. The authors further speculate that this is due to a PTRH2/AES/TLE1 tri-complex; although that has not yet been demonstrated. Additionally, TLE family member 1 (TLE1) functions as a co-repressor with other proteins of the TLE family to regulate transcription and these may also be affected^28,29^. Nuclear TLE1 expression inhibits PTRH2-mediated anoikis by reducing the formation of a cytoplasmic PTRH2-AES complex by keeping AES in the nucleus. Complexing and sequestration of AES by TLE1 in the nucleus, in addition to integrin ligation, are the only known mechanisms that counteract anoikis induced by cytoplasmic PTRH2^30^.

### Localization of PTRH2 to the cytoplasm is regulated by protein kinase D

Signaling activated by ECM-detached integrins upstream of PTRH2 remains poorly understood. However, detachment of integrins leads to PTRH2 cytoplasmic localization and activation of anoikis. There, PTRH2 is phosphorylated at two serines (5 and 87) by activated serine/threonine-protein kinase D (PKD1)^25^. Activated PKD1 is enriched in cells detached from ECM. It is unknown if this phosphorylation of cytosolic-PTRH2 enhances anoikis signaling, however, the authors hypothesize that PTRH2 phosphorylation at serine 5, within the MLS, may block its mitochondrial recruitment keeping PTRH2 in the cytosol^25^. In a similar fashion, the mitochondrial translocation of Cofilin or 2′,3′-cyclic nucleotide-3′-phosphodiesterase (CNP2) is blocked by phosphorylation of serine residues within their MLS sequences^31,32^. This may be due to the conversion of serine to a more acidic phosphoserine, which disrupts mitochondrial translocation^33,34^.

### PTRH2 functions in stress responses via a secretory pathway

PTRH2 can be localized to the endoplasmic reticulum (ER) and Golgi apparatus^4^. In the Golgi, PTRH2 regulates ERK signaling in detergent-resistant microdomains, which are sphingomyelin and cholesterol-enriched regions of intracellular membranes^35^. In these microdomains signaling complexes assemble and signal at internal membrane structures as they would at the plasma membrane^36^. Similar to the mitochondrial-cytosolic model of PTRH2-mediated anoikis, PTRH2 in the Golgi coincides with dephosphorylated ERK1, thereby reducing ERK survival signals and decreasing cell viability. However, if PTRH2 is trapped at the ER and prohibited from progressing to the Golgi, ERK survival signaling is activated due to specific enrichment of phosphorylated, active MAPK pathway components such as BRAF, MEK1/2, and ERK1/2. Cells with PTRH2 constitutively localized to the ER showed significantly increased adhesion-independent viability^4^. PTRH2 exists as an SDS resistant 28 kD dimer at the ER and a signaling-active 16 kD monomer at the Golgi. PTRH2 is also suspected to be associated with the membrane of the ER and Golgi with its C-terminal catalytic domain-oriented in the cytoplasm. This allows PTRH2 to complex with ERK1 localized to the exterior of the Golgi where it disrupts ERK signaling. Furthermore, when ER stress is induced by tunicamycin treatment, this ERK1-PTRH2 complex dissociates and expression of PTRH2 is induced^4^. This pathway is functionally relevant in the organogenesis of the lens and retina. PTRH2 and PKD1 are both expressed differentially in lens fibroblasts and astrocytes during eye development. Retinal astrocytes undergo anoikis during the remodeling of the developing eye. This is mediated, in part, by Golgi-localized PTRH2 inhibiting ERK signaling. In rats with a mutation of a gene encoding βA3/A1-crystallin, eye organogenesis is dysregulated due to aberrant lens fibroblasts and retinal astrocyte signaling. In lens fibroblasts, this mutation prevents normal de-nucleation required for a transparent lens^37^. In astrocytes, it blocks PTRH2 from trafficking to the Golgi, thereby upregulating survival signaling and hindering essential anoikis^38^.

The only eye defects reported in IMNEPD patients were myopia in three sisters with the PTRH2 missense mutation (p.Q85P). Cerebellar atrophy was reported in IMNEPD patients with the PTRH2 truncation mutation (p.Ala90fs and p.W108*) and ataxia and cerebellar hypoplasia was observed in the *Ptrh2*-null mice^8,11^. Potentially, these neurological phenotypes may be caused by deregulation of anoikis or loss of pro-survival signaling in cerebellar astrocytes harboring a PTRH2 mutation because anoikis in astrocytes is essential in the developing cerebellum^39^.

## PTRH2 has regulatory roles in diverse cellular processes

In addition to its well-studied role in pro-survival and anoikis signaling, there is evidence that IMNEPD phenotypes are also caused, in part, by deregulation of cell size, myogenesis, dystrophin complex signaling, and epithelial-mesenchymal transition (EMT; Fig. 2).

### PTRH2 regulates cell size via the mTOR pathway

Histology on tissues isolated from *Ptrh2*-null mice demonstrates significantly smaller cross-sectional areas in neurons, myocytes, exocrine pancreatic acini, and hepatocytes compared to age-matched littermate controls^8^. These findings correspond to cerebellar atrophy, skeletal myopathy, exocrine pancreas insufficiency, and liver pathologies reported in IMNEPD patients. Additionally, ribosomal protein S6 (RPS6) is decreased in *Ptrh2*-null mouse brain and IMNEPD patient fibroblasts, suggesting that abnormal cell size may be due to decreased activity of the mechanistic target of rapamycin (mTOR) pathway^8^. It may be that PTRH2 regulates mTOR through its activation of the FAK-PI3K-AKT pathway because, in the context of cell size, mTOR is activated by way of PI3K/AKT^40–43^.

### PTRH2 regulates myogenic differentiation and maintains skeletal muscle homeostasis

Distal muscle weakness is one of the most prevalent phenotypes documented in IMNEPD patients and in the *Ptrh2*-null mice. Most of the patients suffer from walking gait abnormalities due to severe joint contractures and are wheelchair dependent^8,10,12^. In *Ptrh2*-null mice, severe muscle wasting and skeletal myopathy occur^44,45^. Indeed, *Ptrh2* loss of function severely deregulates myogenic differentiation and disrupts essential integrin signaling in skeletal muscle^3,8,44,45^. PTRH2 levels progressively increase with skeletal muscle differentiation^44^. In myoblasts isolated from the gastrocnemius of *Ptrh2*-null mice, differentiation occurs prematurely due, in part, to a decrease of Bcl2 protein expression, thus significantly increasing the activity of caspases 3 and 9. Notably, during myoblast differentiation, these pro-apoptotic caspases have a non-death function where activated caspase-3 proteolytically cleaves kinases such as serine/threonine kinase 4 (STK4; MST1) or homeodomain interacting protein kinase 2 (HIPK2)^46–48^.

*Ptrh2-null* myoblasts exhibit earlier and higher expression levels of troponin T, myosin-2, and myogenin compared to age-matched controls, suggesting premature differentiation occurs when PTRH2 protein is absent. Re-expression of PTRH2 in null myoblasts rescues early differentiation and decreases caspase activity similar to littermate age-matched controls^44^.

Doe et al.^45^ demonstrated that *Ptrh2*-null myofibers are not only hypotrophic but also exhibit pathological features of congenital muscular dystrophy. Muscular dystrophies are caused by mutations in proteins that comprise the dystrophin protein complex (DPC) that anchors the cytoskeleton underlying the sarcolemma to the ECM and scaffolds signaling proteins. Constant deregulation of signaling at the DPC and its mechanical support of the sarcolemma leads to oxidative stress, which increases myofiber permeability and damages the sarcolemma. The damaged myofiber is lost to necrosis and is replaced via regeneration by satellite cells^49–53^. *Ptrh2*-null mice suffer myofiber damage, which was shown by increased creatine kinase activity and permeability to Evans blue dye. Active regeneration of *Ptrh2*-null muscle fibers was also identifiable by a significant increase in central nuclei, which indicates muscle damage. These mice displayed significant increases in eMyHC expression and endomysial fibrosis; hallmarks of muscular dystrophy^45,53–58^.

The α7β1 integrin is required for healthy muscle development and loss of α7β1 integrin causes Duchenne’s muscular dystrophy in humans and mice^59^. This integrin binds to laminin within the ECM and functions in the regulation of mechanotransduction, hypertrophy, and survival by signaling through AKT, mTOR, and ERK^60–62^. PTRH2 and α7β1 integrin form a complex at the sarcolemma of myofibrils. Interestingly, the ablation of PTRH2 results in a decrease of α7 integrin expression suggesting a feedback loop. *Ptrh2-* and *α7*-null muscle have decreased PI3K-AKT activation indicative of diminished survival signaling via a FAK-PI3K-AKT pathway downstream of α7β1 integrin^45^.

Muscular dystrophy is studied in three different mouse models each representing different deficiencies of DPC components: α7-deficient, laminin-α2 *dyW* null, and dystrophin-deficient *mdx*. PTRH2 expression and AKT signaling has been found to be decreased in *α7*-deficient and *laminin-α2 dyW* null mice but increased in dystrophin-deficient *mdx* mice^45^. In mice, the ablation of PTRH2 alone produces a more severe and lethal myopathy similar to the muscular dystrophy phenotype found in the double knockout of *dystrophin* and *integrin α7β1*^61^. This suggests a pivotal role for PTRH2 in skeletal muscle development.

### PTRH2 regulates epithelial-to-mesenchymal transition (EMT) in lens epithelial cells

Following cataract surgery, lens epithelial cells (LECs) at the anterior capsule of the lens differentiate into a fibroblast-like morphology. This process has been shown in vitro and in vivo to be caused by transforming growth factor-beta (TGFβ) and fibroblast growth factor 2 (FGF2). LECs post-surgery display increased migration and deposit an excess of ECM, which occludes the pre-operative lens^63,64^. This process is mediated in, part, by PTRH2 that is expressed at high levels in the cytoplasm of human LECs. When PTRH2 protein levels are knocked down in LECs, EMT is inhibited despite treatment with TGFβ2 as shown by decreased alpha-smooth muscle actin and migration. TGFβ2 increases PTRH2 mRNA and protein levels in human LECs^65^. Myopia was documented in three patients with the PTRH2 missense mutation (p.Q85P)^11^. Moreover, EMT is an essential process in neural development that is severely dysregulated in IMNEPD patients^66,67^.

## PTRH2 in cancer

PTRH2 has multiple pro-oncogenic and metastasis suppressing functions due to its bi-functional roles in pro-survival signaling, anoikis, and EMT. Due to the heterogeneity and genetic instability of cancers, multifunctional proteins such as PTRH2 may act differently on the available pathways^68,69^. For example, while the loss of PTRH2 expression in cancers of the lung and breast suppresses anoikis, it is upregulated in late-stage esophageal squamous cell carcinomas, perhaps because of its potent cell survival function^30,70–72,57,58,73^. This section will highlight the outcomes of PTRH2 signaling in specific cancers.

### PTRH2 is a regulator of metastasis and EMT in lung and breast cancers

Metastasis is the process by which a primary tumor cell disseminates to distant sites in the body through the lymphatic or circulatory systems^74^. This requires suppression of anoikis pathways and activation of pro-survival pathways, independent of ECM attachment^75^. EMT is a hallmark of metastasis whereby tumor cells become more motile, invasive, and fibroblastic in shape^70,76,77^.

In the context of cancer, PTRH2 has been identified as a potential metastasis suppressor via its regulation of anoikis and EMT. In breast cancer, higher-grade breast carcinomas positive for lymph node metastases stain the least for PTRH2 compared to less invasive breast cancers or normal breast tissue. Additionally, knockdown of PTRH2 protein expression in the breast cancer cell line, MCF7 decreased anoikis sensitivity, increased cell adhesion, and decreased ERK phosphorylation. In vivo, xenograft tumors from MCF7 cells with decreased PTRH2 protein expression did not differ in size or growth rate compared to control tumors. However, metastasis to the lungs increased in the MCF7 tumors with knockdown PTRH2. These tumors contained higher levels of phosphorylated ERK1/2^78^. These results suggest that loss of *PTRH2* expression confers a metastatic advantage in invasive breast cancers.

In lung cancer, a signaling axis containing PTRH2, ERK, and TLE1 prevents EMT. The depletion of PTRH2 protein levels in lung cancer cells (A549 and BEAS-2B) increases migration and vimentin expression but decreases E-cadherin expression thereby promoting increased metastasis in vivo. Overexpression of PTRH2 increases E-cadherin expression and decreases motility and metastasis in vivo. These changes occurred in cells where cytosolic-localized PTRH2 protein was overexpressed; suggesting that cytosolic-PTRH2 regulates EMT. In agreement with previous findings in breast cancer, overexpression of cytosolic-PTRH2 promotes the degradation of TLE1. In detached conditions, cytoplasmic PTRH2 may direct TLE1 to be degraded by the proteasome by an unknown mechanism^30^. This would prevent TLE1 from being recruited to a repressor complex to inhibit E-cadherin expression^57,73^. Furthermore, the regulation of E-cadherin by PTRH2 and TLE1 also involves ERK. In cells with knockdown of PTRH2 protein and treated with an ERK inhibitor, TLE1’s ability to repress E-cadherin transcription was inhibited compared to controls. This suggests that in normal lung tissue, PTRH2 maintains E-cadherin expression by inhibiting ERK. However, in cancer cells with knockdown of PTRH2 protein expression, EMT is driven by active ERK inducing TLE1 repression of E-cadherin transcription^79^.

### PTRH2 is downstream of estrogen receptor and signals through a PI3K/AKT pathway in ovarian cancer

In ovarian serous papillary adenocarcinomas, low PTRH2 protein expression significantly correlates with higher histologic grade and poorer clinical prognosis. In vitro, cytosolic-PTRH2 protein overexpression in Caov-3 cells decreases cell viability^80^. PTRH2 is also detected in sera of patients with serous papillary adenocarcinomas^81^.

As previously described, PTRH2 functions on an integrin-PI3K/AKT pro-survival pathway^8^. In suspended ovarian cancer cells, estrogen (E2) binding to its estrogen (E2) binding estrogen receptor alpha (ERα) blocks the anoikis-initiating release of PTRH2 from the mitochondria and FAs to the cytoplasm through a PI3K/AKT pathway. Cells co-treated with E2 and an AKT inhibitor (CCT128930) have higher cytoplasmic levels of PTRH2 compared to cells with intact PI3K/AKT signaling. This suggests that PTRH2’s translocation to the cytoplasm is inhibited by signals from active AKT^82^. This points to a potential feedback mechanism in which AKT, activated downstream of pro-survival PTRH2-FAK-PI3K signaling, blocks FA and mitochondrial PTRH2 translocation to the cytoplasm.

### PTRH2 supports metastasis in esophageal squamous cell carcinomas (ESCC)

Up until this point, PTRH2 has been discussed for its role in metastasis suppression. However, based on its function in adhesion mediated pro-survival signaling, PTRH2 also acts as an oncogene in specific cancers^58^. Fan et al. analyzed patient-derived esophageal squamous cell carcinomas (ESCC) tissues for differential expression of PTRH2, AIF, and Bcl2 with respect to patient gender, age, and TNM classification, which assesses ESCC extent based on primary tumor size (T) and metastasis to lymphatics (N) or distant sites (M). PTRH2 protein expression is significantly upregulated in ESCC tissues compared to dysplastic and normal tissues. Specifically, higher PTRH2 protein expression correlates with invasive ESCCs, which are poorly differentiated and lymph node metastasis-positive (TMN stages III-IV).

PTRH2 protein expression also correlated with significantly increased Bcl2 and AIF protein levels^72^. Upon the shRNA knockdown of PTRH2 protein in ESCC cell lines (EC9706 and TE1), cell proliferation, migration, and invasion were significantly downregulated compared to controls. Supporting its pro-survival role, knockdown of PTRH2 protein levels increased anoikis. In an in vivo xenograft mouse model, knockdown of PTRH2 protein expression in EC9706 cells decreased tumor size, Bcl2 and matrix metalloproteinase-2 (MMP-2) expression, and increased anoikis^58^.

The oncogenic role of PTRH2 in ESCC may be partly explained by PTRH2’s activation of Bcl2 expression downstream of integrin ligation and PTRH2’s interaction with and activation of FAK^3^ and its ability to activate a FAK-paxillin pathway^58^. As an adaptor protein of FAK, paxillin is required for FAK activation and localization to FAs. Paxillin allows FAK to engage in pro-survival signaling and regulate migration^83,84^. Griffiths and colleagues showed PTRH2 is in a complex with FAK at FAs and PTRH2 may have a role in maintaining FAK-paxillin signaling at the transcriptional level^3,58^. In this ESCC study, microarray data revealed a multitude of differentially expressed genes between PTRH2 knockdown cells and controls. Many of these genes regulated functions where PTRH2 is involved namely cell adhesion, interactions with integrins and myogenesis including Wnt, TLE1, Talin, FAK, and Paxillin^58^.

### A peptide derived from PTRH2 as potential cancer therapeutic

Chen et al. identified a small peptide fragment from the MLS region of PTRH2 that has potential as cancer therapeutic by promoting cell death. The authors called this peptide the PTRH2-Cell Death Domain (PTRH2-CDD). PTRH2-CDD inactivates PTRH2’s pro-survival function and promotes cell death in a caspase-independent manner. It decreased breast cancer cells (MCF-10CA1a) viability in vitro. MCF-10CA1a is an aggressive breast cancer cell line containing a *PIK3CA* H1047R activating mutation that consistently develops tumors in vivo^85^.

Moreover, intra-tumoral injections of iRGD-PTRH2-CDD significantly reduced weight and volume of MCF-10CA1a xenograft tumors and PTRH2-CDD was found in the tumor niche^19^. The PTRH2-CDD peptide contains a species-conserved transmembrane sequence between residues 14 to 33^3,4^. The ablation of this sequence increased PTRH2’s localization to the perinuclear region of the cytoplasm. On the other hand, expression of the PTRH2 catalytic domain alone significantly increased cell survival in HEK 293T cells compared to the expression of full-length PTRH2^19^. IMNEPD patient cells with catalytic domain mutations display reduced cell viability that is rescued by the expression of wild type PTRH2^8^. These findings point to the catalytic domain as a pro-survival region of PTRH2; however, it remains to be determined if the enzymatic activity of this domain is required. Moreover, the PTRH2-CDD peptide exemplifies the bi-functional role of PTRH2. PTRH2-CDD is unable to localize at the OMM and is retained in the cytoplasm where it presumably complexes with GRO/TLE to sequester TLE and thereby promote cell death. Conversely, the PTRH2 catalytic domain alone has a pro-survival effect potentially through an unknown complex at the OMM that may block PTRH2 release into the cytoplasm and subsequent anoikis. The molecular mechanism of how PTRH2 functions as both a pro-survival and a pro-anoikis regulator is an area of an ongoing investigation. It will be helpful to dissect the role of PTRH2 at the OMM to determine how its function at the mitochondria promotes cell survival and blocks anoikis-induction.

## Conclusions

PTRH2 has a crucial role in pro-survival and anoikis signaling decisions. It is an essential participant in integrin-mediated pro-survival signaling pathways in response to adhesion, which allows the cell to make life and death decisions based on specific environmental cues. In cancer, PTRH2 promotes cell survival and metastasis or cell death depending on cancer and signaling context. In human development, PTRH2 provides a cell survival role and is an important regulator of muscle differentiation whereby loss of PTRH2 function causes congenital IMNEPD due to loss of pro-survival signals. PTRH2, therefore, has great potential to be developed as a biomarker and therapeutic target for developmental disorders and specific cancers. To facilitate this, further work is needed to better define the mechanisms that regulate the localization of PTRH2, how PTRH2 activates PI3K and regulates TLE function, and whether enzymatic activity of the catalytic domain is essential to its function.
